# An investigation of the Sustainability of Village Savings and Loans Associations (VSLAs) amidst Covid-19 and its impact on household income levels: lessons from Malawi, Sub-Saharan Africa

**DOI:** 10.1186/s12889-022-13303-9

**Published:** 2022-05-31

**Authors:** George N. Chidimbah Munthali, Xuelian Wu, Mastano Nambiro Woleson Dzimbiri, Amon Zolo, John K.B Mushani, Lazarus Obed Livingstone Banda

**Affiliations:** 1 School of Economics and Management, Yangtze University, Jingzhou, Hubei China; 2 Finance Department, Mzuzu University, Luwinga, Mzuzu Malawi; 3 Chidimbah Research Centre, P O Box 20013, Mzuzu, Malawi; 4 College of Education, Health and Society, Miami University, Oxford, USA; 5 Ministry of Education, Nalikule College of Education, Box 40680, Lilongwe 4, Malawi

**Keywords:** Covid-19, VSLAs, Developing Economies, Inequality of Income, Households Income

## Abstract

**Background:**

Food security, malnutrition, and poverty are some of the challenges that most of the sub-Saharan African countries have been historically facing. With the coming of Covid-19 pandemic, the sustainability of the Village Savings and Loans Association which are formed to counter fight these challenges is questioned.

**Aim:**

This study aimed to assess factors associated with the Sustainability of VSLAs amidst Covid-19 and its impacts on households' income levels.

**Methods:**

An online cross-sectional design was conducted from November to January 2021, targeting VSLAs members in Mzuzu. A snowball and respondent-driven sampling technique were used to recruit the needful participants using a referral approach. IBM SPSS version 23 was used to perform descriptive statistics, Chi-Square, and binary logistic regression with unstandardized Beta (β), Odds Ratios (OR), and 95% Confidence Interval (CI) being taken into account with *P*-value set at 0.1, 0.05 and 0.01 significance levels.

**Results:**

Our study finds that household income declined by 54% for those earnings belonged to ˂ MK5,000, as compared to 38% and 15% for medium (MK5,000 ≥ MK10,000) and higher (> MK10,000) income bands respectively. Our study shows that gender (β = 0.437, *p* = 0.094), age-group (β = 1.317, *p* = 0.000), education (β = 2.181, *p* = 0.047), share contributions (β = 1.035, *p* = 0.008), meetings (β = 0.572, *p* = 0.021), occupation (β = -0.453, *p* = 0.106), and frequency of meeting (β = -0.507, *p* = 0.049) were positively and negatively statistically significant predictors.

**Conclusion:**

According to the findings of this study, households with lower income earners, which is one of the indicators of poverty, are more affected by the pandemic than their counterparts. We urge that the Malawi governments should maintain and, if they haven't already, implement programs that support low-income households, such as transfer payments, which have been shown to uplift people out of income poverty in many developing countries.

## Background

The outbreak of Covid-19 in 2019 in the city of Wuhan in Hubei Province in the People's Republic of China came with rapid disastrous effects globally [1–3]. The virus is said to cause respiratory syndrome, and people with prevalent underlying health conditions are more prone to the infection [4–7]. According to public health experts, the human-to-human viral transfer has caused many deaths as well as considerably negative impact on the economic, social, and political activities globally [8–12]. Scholars have written on how Covid-19 impacts the global economies such that no single country has been spared from its fate [4, 9, 13–15].

Over the past decades, there has been a proliferation of groups of people with common economic targets and desires to generate and save money among themselves. More than two people with common interests come together to form a Village Savings and Loans Associations (VSLAs) group with the aim of saving, borrowing, and even making insurance funds in the form of shares that they buy at the beginning and act as the amount of contribution per defined period. Countries across the globe have different terms for these Village Savings and Loans Associations (VSLAs), irrespective of the groups bearing the same goals [16]. For instance, in Egypt, they are called Rotating Savings and Credit Associations (ROSCAs) or, Accumulated Savings and Credit Associations [17, 18]. Loans obtained from these associations are of low interest compared to other microfinance banks. Many of these VSLAs are not registered with the registrar of the business. They usually act on a trust basis since they just unite people from the same village, workplace, church, or any other common ground. Usually, the activity runs for a cycle of one year or so. After the end of the cycle, the members share the dividends themselves [19, 20].

VSLAs have for the past decades or so proven to be one of the remarkable tools, platforms, modes, or drivers of economies worldwide, which have positively impacting the living standards of the rural dwellers, especially in developing countries [21, 22]. For instance, out of 176 of the significant and most sustainable Micro Finance Institutions (MFIs) found in seventeen Latin American countries, forty-seven of them are into village banking [23]. Besides, the literature indicates that in African countries like Ghana, Malawi, Rwanda, Kenya, Sierra Leone, Egypt, Ethiopia, Uganda, Nigeria, where CARE international and VSLAs Associates implement their programs, similar impacts of VSLAs on members' lives and the economy, in general, have manifested [21, 24–26]. More importantly, the VSLAs have significantly promoted gender equality by empowering women through economic decision-making at household and group levels [18, 21, 27]. Further, the program has helped to reduce or eradicate poverty by increasing the disposal income to individual members as well as increasing agricultural productivity through loans, which enable them to afford farm inputs throughout the cycle [24, 28, 29]. Besides, the VSLAs have helped reduce maternal health problems in developing countries as women can access instant loans to pay for convenient transportation to seek medical relevant attention [26].

While appreciating all the positive impacts of VSLAs, it is worthy to note that the spread of Covid-19 has also affected the operations of these VSLAs, which has further challenged its sustainability. Despite the voluminous literature about the impact of Covid-19 on people's lives, there is little systematic documentation as to how the pandemic has impacted the Village Savings and Loans Associations (VSLAs) performances among residents in developing countries like Malawi. Our study fills this gap by examining the socio-economic status and sustainability of VSLAs. We achieved our goal by answering the following research questions: (i) What is the impact of covid-19 on household income levels (ii) What factors determine the sustainability of VSLAs?

## Methods

### The aim

This study aimed to assess factors associated with the Sustainability of VSLAs amidst Covid-19 and its impacts on households' income levels.

### Study design

**Fig. 1:**
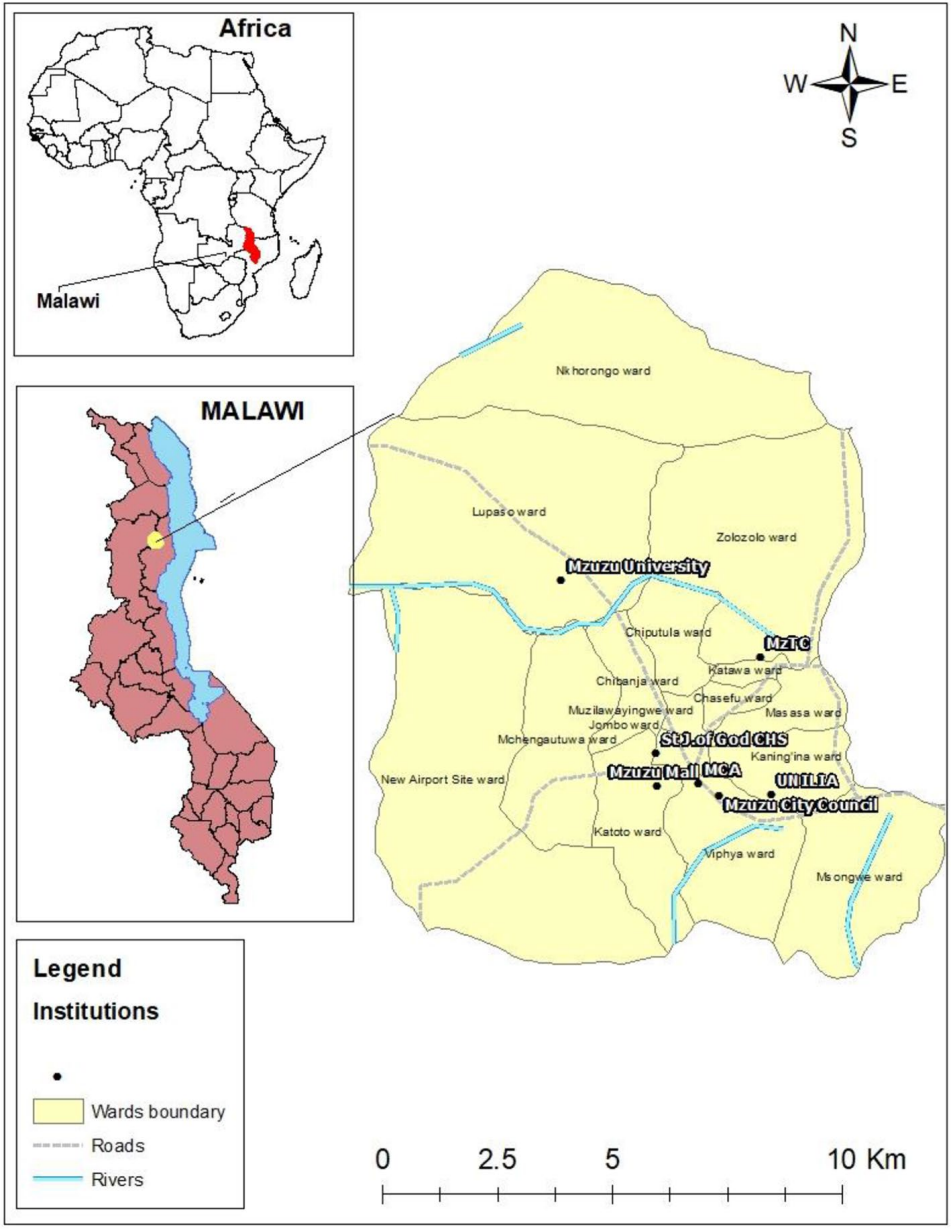
Study Area of Mzuzu. Source: Authors 2021

This study used a quantitative method design to extrapolate in-depth information about the topic under study [30, 31]. In this study, we used an online cross-sectional survey. The study was conducted in Malawi, Africa. Malawi is located in the southern part of Africa, bordering countries like Zambia, Tanzania, and Mozambique [32, 33]. It is one of the least developing countries in the world where a majority of its population still faces hunger, malnutrition, and lives under the poverty line [15, 34, 35], and depend mostly on agriculture for business and feeding the population [36]. In Malawi, the study was specifically conducted in Mzuzu city, which is located in the northern part and the city has an estimated total population of 221, 272, and a land area of 143.8 square kilometers as of 2019. The study area is politically divided into 14 wards, namely; Chibanja, Chibavi West, Chibavi East, Chiputula, Jombo-Kaning'ina, Katawa, Lupaso-Nkhorongo, Luwinga, Masasa, Mchengautuwa East, Mchengautuwa West, Mzilawaingwe, Zolozolo East, and Zolozolo West) as shown in Fig. 1. According to recent research, Mzuzu City is one of Malawi's fastest-growing cities, primarily to its social-economic activity, with most of its inhabitants dependend on agriculture, business, and working as civil servants in many of the city's governmental and non-governmental organizations [37]. Given these conditions, it was critical to conduct this study in this city to examine the impacts of Covid-19 that might have a negative influence on the people's socio-economic status.

### Data collection procedures

We collected data from various VSLAs members operating within Mzuzu City. The data collection exercise was done between November 2020 and January 2021. We recruited and trained a team of four research assistants, who were guided on the aims and how to conduct the study. These research assistants helped to identify VSLAs groups and their members and facilitated the collection of the online survey headed by Mr. Zolo (as head of the Research assistants). The questionnaire was sent to the participants using Facebook, WhatsApp, and email due to the Covid-19 gathering restrictions, as the study was conducted when some social distance measures were being enforced in Malawi. The targeted respondents identified were forwarded the questionnaires links with assistance from the recruited research assistants.

### Inclusion and exclusion criteria

All members of VSLAs that were operating within Mzuzu localities, who were below 18 years old were excluded from the survey. To ensure that the respondents were from that country and city, a space was provided in the questionnaire instrument where the respondent stated their country and city of residence. All those who indicated outside the study areas were excluded.

### Population, sample size, and technique

The study recruited the respondents that were members of VSLAs in the designated study area. A snowball and respondent-driven sampling technique was used to select the area and determine the sample population. We employed this technique based on the following reasons. First, it was easy to access data due to the researchers' connections with people associated with VSLAs in the selected country. Second, due to the impact of Covid-19, it is not easy to collect the data physically, taking into consideration the social distance measures in place in all countries [38–40].

We recruited 402 participants in the survey based on the inclusion and exclusion criteria. We employed a sampling calculation used by Yamane, with a 95% Confidence Level and, *P* = 0.05 [41], *N* = Total Population of Mzuzu City = 221,272 [37].

$$x=\frac{N}{1+N{(e)}^{2}}$$

Which gave us 399, as the minimum required number of participants.

### Questionnaire' design

The questionnaire had three sections.

i. **Demographic data**
  The first sections captured the social demographic data of the VSLAs members which include; the respondent's gender, age group, occupation, education level, the status of the household head, and the number of people in the house of which twere measured in the category, and coded in binary form (Table 1).
ii. **Impact of Covid-19 on Income **
  The second part of the questionnaire captured data on the impact of Covid-19 on the income of the participants. We asked the respondents to indicate the category of income that they earn per month before and during the outbreak of Covid-19. The income was put in categories/groups of three income bands or levels. The first one was those falling under Less than MK5,000, then those under or above MK5,000 but less than MK10,000, and finally those above MK10,000 (Table 1).
iii. **Performance and sustainability indicators**

**Table 1:** Variables definitions and coding

Variables	Category	Categorical Variables Coding	
		**Frequency**	**Parameter coding**
Gender	Male	259	0
	Female	142	1
Age-group	< 30	157	0
	≥ 31 years old	244	1
Occupation	Otherwise	288	0
	Employed i.e. Civil servant or NGOs	113	1
Education Level	otherwise	14	0
	attended education	387	1
Are you head of the house	No	122	0
	Yes	279	1
Number of people in the house	˂5 members	139	0
	6 ≥	262	1
Loan Repayment not on time	No	77	0
	Yes	324	1
Loan obtainment Frequency	Decreased	274	1
	Increased	127	0
Shares contribution not time	No	89	0
	Yes	312	1
Meeting’s continuations	No	160	0
	Yes	241	1

Source**:** Authors 2021 Coded Using SPSS Software

The third part of the questionnaire captured the data on the performance which predicted the sustainability of the VSLAs amid Covid-19 based on the literature. Members were asked questions regarding the impacts of Covid-19 on; loan repayment in time, loan obtainment frequency, shared contributions in time or not, and if the members were meeting. All variables were categorical and were measured in binary form (Table 1).

### Validity and reliability

A pilot study was conducted to pre-test the instruments before the actual collection of data which involved 43 respondents comprising VSLAs members, Masters’ and Ph.D. students. The validity and reliability of the instrument were tested by sending the instrument to experts for comments before actual data collection. The research instrument was tested by using Cronbach’s Alpha in SPSS and was found to be 0.8.

### Ethical clearance

Ethical clearance of this study was reviewed and approved by the School of Economics and Management of Yangtze University (Approval number REF/YU/2020/08 (Fig. 3)) and Mzuzu City Council (Approval letter reference number MCC/dated on August 12, 2020 (Fig. 4)). Further, the researchers observed and followed the 1964 Helsinki Declaration under conducts of research involving human beings. Participation in the survey was voluntary and the participants gave their informed consent to this questionnaire before completion.

### Data analysis

After collecting the data using the google form, we coded it in Microsoft Excel and later imported it into SPSS Version 23 for analysis. We presented the results of the descriptive statistics using frequency tables, graphs, and charts. The Chi-Square test was performed to determine associations between socio-demographic variables and other variables. *P-value* was statistically significant at *p* < 0.05. Lastly, due to the nature of our dependent variables, a binary logistic regression model was performed.

### Econometric model specification

This was used to address our second research question: to predict the factors associated with the sustainability of VSLAs. We used Certainty of Future of VSLAs as our dependent variable, which was coded or characterized as a two-categorical variable. The coding was that if the VSLAs members attest that they have certainty in the future and sustainability of VSLAs regarding the current situation of Covid-19, then the value given was 1; otherwise, 0. Therefore, the dichotomy of the dependent variables directed and suggested to us used the binary logistic regression model, which was deemed fit as used by other scholars[14, 42, 43].

In this study, a logistic regression model, the dichotomous variable is defined as,

$$y={\int }_{0}^{1}whereas 1=The Presence of the Characteristics 0=The absence of the Characteristics$$

whereas the odd is defined as,

$$Odds=\frac{p}{p-1}=\frac{The Probability of the Presence of the characteiricts}{The probability of the absence of the characteristics}$$

whereas the definition of the logit model is like this,

$$Logit\left(p\right)={\beta }_{0}+{\beta }_{1}{X}_{1}+{\beta }_{2}{X}_{2}+{\beta }_{3}{X}_{3}+\cdots +{\beta }_{k}{X}_{k}$$[43, 44]

Where the probability of the presence of the characteristic of interest is represented with p. The logit transformation is defined as the log of odds.

$$\mathrm{log}\left(\frac{p}{1-p}\right)=Logit\left(p\right)={\beta }_{0}+{\beta }_{1}{X}_{1}+{\beta }_{2}{X}_{2}+{\beta }_{3}{X}_{3}+\dots +{\beta }_{k}{X}_{k}+e$$[43, 44]

Whereas;

$${\beta }_{0}$$= Constant,

${\beta }_{1}-{\beta }_{k}$= are the coefficients of logistic regression,

${X}_{1}-{X}_{k}$= are independent explanatory variables, and.

$e$= is an error term.

## Results

The results of this study reveal that a total number of 402 VSLAs members participated in responding to the survey representing a 100% plus after meeting a target of 399, which was found using the sampling techniques (Table 2).

**Table 2:** Social demographic characteristics and Future of VSLAs (*N* = 402)

Variables	Category	Future of VSLAs Is		n	%	χ2 Test	*P*-value
		**Certain**	**Not Certain**				
Gender	Male	131	128	259	64.4279	4.462	.035**
	Female	88	55	142	35.3234		
Age group	< 30	56	101	157	39.0547	36.749	.000***
	≥ 31 years old	163	82	244	60.6965		
Occupation	Otherwise	152	137	288	71.6418	1.469	0.225
	Employed	67	46	113	28.1095		
Education Level	otherwise	1	13	14	3.48259	13.105	.000***
	attended education	218	170	387	96.2687		
Head of the house	No	118	65	122	30.3483	4.249	.039**
	Yes	162	57	279	69.403		
Household Total (Pple)	˂ 5 members	76	63	139	34.5771	0.003	0.954
	6 ≥ members	143	120	262	65.1741		

Source: Authors 2021

^*^Significance at 10%

^**^Significance at 5%

^***^Significance at 1%

*Abbreviations***:** *n* Total Frequency, *%* Percentage, *χ2* Chi Square Test, *apple* People

### Socio-demographic characteristics and future of VSLAs amidst Covid-19

Table 2 shows that the majority of the respondents were male (64%, *n* = 259) and were statistically significantly associated with the certainty of the future of the VSLAs (χ2 Test = 4.462, *p*˂0.05). Most of these were above 30 years of age (60%, *n* = 244), which was also statistically significantly associated with the VSLAs (χ2 Test = 36.75, *p < *0.05). The majority of these were not formally employed either in government or NGOs 71% (*n* = 288), despite that they attained a certain level of education 96% (*n* = 218) and was also statistically significantly associated with the certainty of VSLAs (χ2 Test = 13.105) *p* < 0.05). Further, the results show that the majority of the respondents were heads of households (69%, *n* = 279) and were also found statistically significantly associated with the certainty of VSLAs (χ2 Test = 4.249, *p*˂0.05). Most of these had also households with an average of 6 members (*n* = 262).

### Impact of Covid-19 on the income of the VSLAs Members

**Fig. 2:**
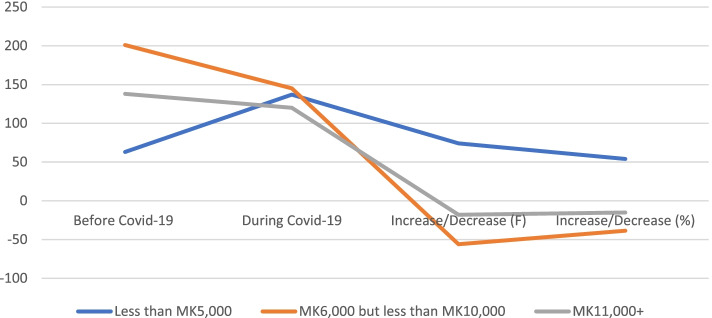
Impacts of Covid-19 on Households Income of VSLAs Members. Source: Authors 2021

Regarding the change in household income, the results show that the majority of the respondents reported a downward shift in earning (lower) income per month of less than MK5,000 with an approximate 54% (*n* = 74) during the pandemic (Table 3 and Fig. 2). This has caused a tremendous negative effect on the medium income band of above MK5,000.00 but less than MK10,000.000 with an approximately -38.62 (*n* = -56) decline, and on the last income band, of above MK10,000.00 also indicated a decline with a lower percentage of 15% compared to the previous income band. Using the ANOVA test, results show statistically significant at *p-*value of 0.05 level of significance with F = 103.705, *p* = 0.000 (Mean = 2.19, SD = 0.7) values thus before and during F = 101.78, *p* = 0.000 (Mean = 1.96, SD = 0.8).

**Table 3:** Impact of Covid-19 on income earnings (Income changes due to Covid-19) among VSLAs members

	**Category**	**Income Bands**			**Mean**	**SD**	**F-Value**	***P*-Value**
		**< MK5,000**	**MK5,000 ≥ MK10,000**	** > MK10,000**				
**Period**	Before Covid-19	63	201	138	2.19	0.7	103.705	.000**
	During Covid-19	137	145	120	1.96	0.8	101.780	.000**
**Changes**	Frequency (n)	74	-56	-18				
	Percentage (%)	54.01	-38.62	-15				

Source: Authors 2021

Changes means changes from (Before to During period of Covid-19) calculated as in frequency (n) and percentage (%)

^***^Significant at the 5% level of significance

(One Way ANOVA). 1 USD = MK811.37

### Predicting the factors associated with the future and sustainability of VSLAs amid Covid-19

We performed a binary logistic regression to predict factors associated with the Future and sustainability (certainty) of the VSLAs (Table 4).

**Table 4:** Predictors of the certainty of the future and sustainability of VSLAs during Covid-19 using by Binary Logistic Regression Model

Variables	β	OR	95% C. I
Gender of the respondent (1)	0.437(0.094)*	1.548	[0.928;2.582]
Age group (1)	1.317(0.000)***	3.732	[2.316;6.012]
Occupation (1)	-0.453(0.106)*	0.636	[0.367;1.101]
Education Level (1)	2.181(0.047)**	8.852	[1.033;75.888]
Are you head of the house (1)	-0.002(0.994)	0.998	[0.6;1.658]
Number of people in the house (1)	-0.174(0.481)	0.84	[0.518;1.363]
Shares contribution not on Time	1.035(0.008)***	0.355	[0.166;0.759]
Frequency of loans obtaining increasing	-0.507(0.049)***	0.602	[0.364;0.998]
Loan Repayment not on time	-0.368(0.372)	0.692	[0.309;1.553]
Meetings	0.572(0.021)***	1.773	[1.09;2.881]
Constant	-1.506(0.211)	0.222	
Model Predicted Success	68.10%		
Log-likelihood ratio	465.102		
Hosmer and Lemeshow Test	(df = 8) significance test result 8.469 (*P*-value = 0.389)		
Omnibus Tests of Model Coefficients	(df = 10) significance test result 87.387 (*P*-value = 0.000***)		
Cox and Snell R2	0.196***		
Negelkerke R2	0.262***		
Sample Number (n)	420		

Source; Authors 2021

*Abbreviation*: *OR* Odds Ratios

(1): Reference Category using/based on Table 2 coding specifications

^*^Significance at 10%

^**^Significance at 5%

^***^Significance at 1%

Our findings revealed that the model fitted the data as the Hosmer and Lemeshow Test was found not-Significant at *P*-value = 0.389 (> 0.05). Further, in this model, we have tried to explain the variance of 19.2 to 26.2% based on Cox and Snell R2 and Nagelkerke R2 values, respectively. The Model further gave a 68.10% Model Predicted Success.

After assessing the explanatory variables inputted in the model, out of 10 variables, seven were statistically significantly and these included gender (β = 0.437, *p* = 0.094), age (β = 1.317, *p* = 0.000), education (β = 2.181, *p* = 0.047), shared contributions (β = 1.035, *p* = 0.008), and meetings (β = 0.572, *p* = 0.021). However, occupation (β = -0.453, *p* = 0.106) and frequency of meetings (β = -0.507, *p* = 0.049) were not significant predictors. On the other hand, household head (β = -0.002, *p* = 0.994), number of household members (β = -0.174, *p* = 0.481), and loan repayment (β = -0.368, *p* = 0.372) were also not significant predictors in the model.

Further, to increase the reliability by not only depending on the values of the coefficients, we also assessed the odds ratios explained as a probability of the VSLAs members being certain about its future based on the literature (Kunst, 2013). Thus, our results revealed that males had 1.55 times (odds ratio [OR]: 1.548; 95% CI: 10.928;2.582) more likely to have certainty in the future of VLSAs than females. Members within the age group of ˂30 were 3 times (odds ratio [OR]: 3.732; 95% CI: 2.316;6.012) more likely to be certain about the future of the VSLAs. Regarding occupation, those who were not employed were found 3.73 times (odds ratio [OR]: 3.732; 95% CI: 0.367;1.101) more likely to be certain about the future of the VSLAs compared to those working. In terms of education status, participants with no education were 8.85 times (odds ratio [OR]: 8.852; 95% CI:1.033;75.888) more likely to be certain about the future of VSLAs compared to educated ones. When asked about the head of a household status, the results show that those who are not head of household were 0.99 times (odds ratio [OR]: 0.998; 95% CI:0.6;1.658) less likely to be certain about the future of VSLAs compared to household heads. Furthermore, our results show households with less than ˂5 members were 0.84 times (odds ratio [OR]: 0.84; 95% CI:0.518;1.363) less likely to be certain about the future of VSLA compared to their counterparts.

Moreover, the results of the odds ratios of the performance indicators have demonstrated that not contributing shares in good time led to 0.36 times (odds ratio [OR]: 0.355; 95% CI:0.166;0.759) more likely to lead to the certainty of the VSLS. Additionally, members having meetings since the outbreak of Covid-19 were found 1.77 times (odds ratio [OR]: 1.773; 95% CI: 1.09;2.881) more likely to contribute to the certainty of the VSLAs compared to not meeting. The results also show that an increase in the frequency of obtaining loans has 0.60 times (odds ratio [OR]: 0.602; 95% CI: 0.364;0.998) less likely to led to the certainty of VSLAs and those who agree on loan repayment not in time was found 0.69 times (odds ratio [OR]: 0.692; 95% CI: 0.309;1.553) less likely to lead to the certainty of the VSLAs in times of Covid-19.

## Discussion

This study aimed at investigating the predictors associated with the sustainability of Village Savings and Loans Associations (VSLAs) and its impact on household income levels amid Covid-19. Our analyses show an increase in male engagement in social associations such as VSLAs, even though these groups are generally perceived to be for women[27]. We could attribute this development to the fact that most members of VSLAs, especially males, are considerably educated and have a positive mindset towards the groupings. However, it is not surprising to see that the majority of the respondents were unemployed despite having good education achievements. Unemployment remains a huge problem in the least developed countries and Covid-19 has further crippled the economies of poor countries. Our findings are consistent with other recent studies conducted by Governments and Non-Governmental Organisational (NGO) such as United Nations International Children's Emergency Fund (UNICEF) in developing countries like Indonesia [45, 46], Kenya [45, 46], and developed countries like the United States of America (USA) [47], and United Kingdom (UK) [48] where they found that a decline in the levels of employment during the Covid-19 pandemic as the vast majority continue to lose jobs. Most of the current members of VSLAs are adults who can also make well-informed decisions on social-economic investments and social capital set-ups. Being ahead of household automatically pushes them to join such savings groups geared to increasing their disposable income so that they can better support their families. Further, in most African cultures, children are considered a source of wealth. Thus, it is not surprising that many households had more than five members, which is different from European, American, and Asian countries, where households have three to four members on average [49, 50]. Studies have shown that large households are probably one of the contributing factors to unemployment, food shortages, poor women's health, and other economic hardships as faced in many developing countries [51, 52].

Our study has further revealed that the outbreak of Covid-19 has negatively impacted the household income of the VSLAs members. It is evidenced that the income of the vast majority of VSLAs members has drastically declined after the outbreak of Covid-19 compared to the period before Covid-19 began. Thus, the Covid-19 measures in place by authorities in many countries such as lockdowns, curfews, and other social distances in combating the severity of the impact of the pandemic have ironically resulted in these negative consequences on people’s business and economic activities [23, 53]. Our findings agree with many recent studies conducted across the world on the impact of Covid-19 on different sectors of economies and household income due to disruption of the supply chain, agriculture, education, sports, health, and transportations [15, 54, 55]. For example, a recent study conducted in Kenya revealed that households’ income levels of those with low earnings had declined pervasively as many family members were declared redundant in their workplaces, there was a reduced rate of getting gifts, and earnings from remittance rates were also reduced due to Covid-19 [46]. Likewise, a study conducted in the USA agrees with our study as they also found a pervasive decline in household income among individuals during the Covid-19 pandemic [56]. Similar results were found in a study conducted in South Africa where income earnings of most households tremendously declined to lead to severe food shortages in many households [57].

Despite experiencing the negative impact of Covid-19, there is a small percentage of the members who earn above MK10,000 per month and a slight change for those who were in this category of the lower-income bands. This is clear evidence that in developing countries like Malawi, the majority of the population is still not earning a higher income either from work or business. This agrees with some studies that found that in Malawi and other developing countries, the vast majority of the populations are still earning lower income and are living under poverty line [58, 59]. In our study, we can also appreciate the impact of income inequality has played on the households coping strategies during the Covid-19 pandemic. Evidence shows that those falling within the lower income levels (band) are more affected compared to those in medium and higher income levels (bands). Our findings agree with other studies that found that inequality income distribution within a society has impacted differently on households in the context of coping and recovery process during the Covid-19 pandemic period [60, 61], which gives the authorities much attention when it comes to policy implementation to cautions the impact of the Covid-19, and other future related epidemics and pandemics, disasters, etc.

Regarding the sustainability of VSLAs, the results from binary logistic regression revealed that some social-demographic characteristics are significant predictors influencing the sustainability of the VSLAs in the times of Covid-19. First, being a male member had a positive impact on the certainty of the VSLAs. Drawing evidence from some physiological studies, it is argued that males are risk-takers and they tend to have a positive perception of something compared to women (ceteris paribus) [62, 63]. Further, this agrees with another recent study conducted in Spain on Covid-19 risk perception which found that females perceived higher dangers of Covid-19 compared to males [64]. Additionally, a study conducted in the United State of America (USA) also found that females were more afraid of Covid-19 compared to males[65]. Age groups of ≥ 31 years old were more likely to lead to the certainty of sustainability of VSLAs, despite the fact that many factors contribute to the maturity of an individual such as age. Thus, the age group of the individuals who have moved out of adolescence and are said to be mature enough has high experience of life uncertainties[66, 67]. In regards to the fact that Covid-19 outbreak was new to be encountered, but this age-group (≥ 31 years old) having and being experienced with of life of developing countries like of Malawi, and some of Africa in general, had at one point in time experienced some of the other deadly health epidemic and pandemic like Cholera, Malaria, Ebola, etc., thus it can be argued that this could at least reduce their fear, and improved their perception [68].

In continuation, education was also found to be a significant predictor of the sustainability of VSLAs amid Covid-19. Thus, the majority of VSLAs members are educated and have a positive perception of the certainty and sustainability of VSLAs compared to uneducated ones. One could argue that education increases one's awareness, information, and knowledge of something gained through online and other channels of communications dissemination. Hence, this made most educated members be well informed and have knowledge of Covid-19, which helped them to have a positive perception of it, like any other pandemic which needs to be fought with positivity. Our findings are supported and in agreement with a recent study conducted on the topic of Knowledge Attitude and Preventative Practices (KAPs) studies on Covid-19 which found a strong relationship with knowledge, attitude, and perceptions of Covid-19 among different classes of people [69–71]. Further, on socio-demographic characteristics, we found that occupation is negatively impacted on the certainty of VSLAs. Thus, members of the VSLs who were employed perceived a lack of certainty and sustainability in the VLSAs. This could be explained by the fact that due to the pandemic there was a lot of job insecurity among employees as many firms were, and are at the center of making massive losses due to business disruption either by lockdowns or other social distance preventative measures of Covid-19 [33, 38].

Furthermore, the study found that delayed contribution of shares by members had a positive impact on VSLAs certainty. Our study disagrees with other studies that suggest that the contributions of the shares add to the certainty, performance, and sustainability of the VSLAs [25, 72]. Several reasons could be explained regarding this disagreement, some being the nature of methodology we used, the environmental factors such as Covid-19, as the other studies were not conducted under similar circumstances. However, an increase in the number of people obtaining loans from the group had a negative impact on the certainty of the VSLAs. Not surprisingly, as we also found in this study that there was a positive correlation of members not repaying loans on time. Meaning that, as the number of people obtaining loans increased, members were unable to repay loans on time which could also pose a threat as they were able to deplete the savings without replenishments. Our study also found that meetings had a positive impact on the certainty of the VSLAs. This means that physical and online meetings of the members were able to give each other social strength, hope, and support in achieving their agenda. This finding is also in line with principles of social organizations, formal or informal. Meetings enabled the members to challenge and energize each other. Our results corroborate prolific scholarly evidence in the field of social capital attesting that social gathering improves the mental, physical and social wellbeing [73]. Furthermore, our findings are also in agreement with other Covid-19 related studies where they found that despite being in times of Covid-19, the availability of online platforms and digital transactions, members of village savings were able to meet and transact their business making them cope with the pandemic [74].

### Limitations

Despite that we have managed to achieve our aim of the study, we faced the following limitations. First, our study was a cross-sectional design, hence it was difficult to analyze causal relationships between study variables. Second, we used an online platform to collect data. As a result, the majority of the respondents had difficulties or faced hardships to find income that would enable them to buy data bundles for the internet, which is very expensive in Malawi. Also, since our study was not funded, it was challenging to convince more people to participate in the study. However, we tried to overcome this limitation by proposing a rotary gift for the group that will have the majority of the respondents.

## Conclusion

This study has found that Covid-19 has negatively impacted the household income of the VSLAs members in Malawi. However, the impact varies based on the level of household earnings. Members of the VSLs with low-income levels are more negatively affected as compared to the medium and the higher income levels respectively. Inequality in income distribution has affected the coping and recovery strategies towards Covid-19 in Malawi. On the other hand, gender, age group, education, shared contributions, and the continuation of meetings were significant predictors associated with the certainty and sustainability of VSLAs. In contrast, occupation and frequency of meetings were not significant predictors regarding the sustainability of VSLAs. Further, head of household, household size, and loan repayment were also not significant predictors using the binary logistic regression model.

### Recommendations

Several lines of evidence have proved useful in helping to predict the impact of Covid-19 on the household income of the VSLAs members in Malawi.

Based on our findings, the study will generate scientific evidence that will serve as an important data source of local, national, and global significance in expanding existing understanding on various local programs and initiatives such as transfer payments, which have been shown to increase income in many poor countries. This will also become an increasingly important tool for promoting gender equality by empowering individual community members through economic decision-making at the household and group levels amid Covid-19.

Additionally, the programs will also help to reduce or eradicate poverty by increasing the disposal income to individual members, increasing agricultural productivity through loans enabling them to afford farm inputs, as well as reducing public health problems such as maternal health amid Covid-19. It will also be beneficial to policymakers and program planners at all levels in reviewing success and targeting future efforts while offering evidence-based public health action to prevent and control probable Factors Contributing to the Fast Spread of the Covid-19 in low-income countries.

Taken together, these findings do not support strong recommendations, however, they can be used for future studies that should be done in Malawi to analyze the impacts of Covid-19 at the Macro level that would be able to try to quantify the impact.

## Data Availability

All data and other materials are included in the manuscript but if anything, apart from the available in the manuscript can be requested from the authors.
